# Effects of Smokeless Tobacco on Color Stability and Surface Roughness of 3D-Printed, CAD/CAM-Milled, and Conventional Denture Base Materials: An In Vitro Study

**DOI:** 10.3390/biomedicines11020491

**Published:** 2023-02-08

**Authors:** Maryam H. Mugri, Saurabh Jain, Mohammed E. Sayed, Amjad Hussain Asiri Halawi, Safa Ahmed Ibrahim Hamzi, Raniya Abdulaziz Saad Aljohani, Zainab Mousa Ali Madkhali, Asaad Khalid, Hossam F. Jokhadar, Mai Almarzouki, Ghaida A. Alhumaidan, Ahid Amer Alshahrani, Saeed M. Alqahtani, Nasser M. Alqahtani, Honey Lunkad

**Affiliations:** 1 Department of Maxillofacial and Diagnostic Sciences, College of Dentistry, Jazan University, Jazan 45142, Saudi Arabia; 2 Department of Prosthetic Dental Sciences, College of Dentistry, Jazan University, Jazan 45142, Saudi Arabia; 3 Rutgers School of Dental Medicine, Rutgers University, Newark, NJ 07103, USA; 4 College of Dentistry, Jazan University, Jazan 45142, Saudi Arabia; 5 Substance Abuse and Toxicology Research Center, Jazan University, Jazan 45142, Saudi Arabia; 6 Medicinal and Aromatic Plants and Traditional Medicine Research Institute, National Center for Research, Khartoum 11111, Sudan; 7 Department of Oral and Maxillofacial Prosthodontics, Faculty of Dentistry, King Abdulaziz University, Jeddah 21589, Saudi Arabia; 8 Department of Operative Dentistry, King Abdulaziz University, Jeddah 21589, Saudi Arabia; 9 King Saud Medical City, Riyadh 12271, Saudi Arabia; 10 Department of Dental Technology, Applied Medical Science, King Khalid University, Abha 62529, Saudi Arabia; 11 Department of Prosthetic Dentistry, College of Dentistry, King Khalid University, Abha 62529, Saudi Arabia; 12 Department of Prosthodontics, College of Dentistry, King Khalid University, Abha 62529, Saudi Arabia

**Keywords:** 3D printing, heat polymerization, CAD/CAM, milling, tabbaq, khaini, smokeless tobacco, color, surface roughness, denture base resin, PMMA

## Abstract

Tobacco consumption in its different forms can affect the optical and surface properties of dental materials that are used in the oral cavity. Thus, the present study was conducted to evaluate the effects of two commercially available smokeless tobacco products on the color stability and surface roughness of denture base resins that were fabricated using three different techniques (CAD/CAM milling, 3D printing, and conventional heat polymerization). A total of 126 denture base resin specimens were fabricated using the three different manufacturing techniques (*n* = 42 each). Specimens from each group were further subdivided into three subgroups (*n* = 14 each) and immersed in three different immersion media (a khaini suspension, a tabbaq suspension, and artificial saliva). The differences in color and surface roughness were assessed according to data that were collected and statistically analyzed using SPSS version 24.0. The tabbaq smokeless tobacco was found to cause greatest changes in color and surface roughness; the effect was observed to be highest in the 3D-printed specimens followed by the conventional heat-polymerized and CAD/CAM milled specimens. The mean changes in color and surface roughness were the highest for the tabbaq smokeless tobacco followed by the khaini smokeless tobacco and the artificial saliva. Statistically significant (*p*-value < 0.05) differences were observed among all techniques and suspensions. We concluded that the mean changes in color and surface roughness were significantly higher for the 3D-printed dentures compared to the conventional heat-polymerized and CAD/CAM-milled dentures. Thus, the results of the present study strengthened the concept that tobacco in any form can lead to changes in the color and surface roughness of denture base materials.

## 1. Introduction

Edentulism is a frequently encountered clinical condition, especially among elderly individuals, that affects the oral-health-related quality of life [1]. In the most recent generational cohort, the incidence of edentulism has decreased; however, with the increased life expectancy, an overall increase in the number of edentulous patients has been observed [2]. Edentulism is a condition that is mainly caused by periodontitis and dental caries. It can be managed through the use of implant-supported prostheses or conventional complete dentures (CDs) [3].

The most commonly used material for the fabrication of conventional CDs is polymethylmethacrylate (PMMA) resin due to its ease in manipulation and handling, low cost, and satisfactory aesthetics, as well as its physical and mechanical properties [4]. However, the main disadvantages of this material include vulnerability to fracture and abrasion, changes in color and dimensions, irritation of tissues, and increased susceptibility to infections [5]. The mechanical properties are hampered by the presence of subsurface voids, which also compromise the aesthetics of the dentures [6].

Recently, digital technology has been used in different dental specialties. The manufacturing and design of dental prostheses with the help of various devices, materials, computer-aided design (CAD), and computer-assisted manufacturing (CAM) have helped to reduce the workload of dental technicians and dentists [7,8]. CAD/CAM denture bases can be milled from a highly compacted acrylic resin or can be printed from photo-polymerized resins. These digitally fabricated materials exhibit lower polymerization shrinkage [8], reduce the number of required appointments, and provide ease of electronic records maintenance [9]. CAD/CAM-milled denture base resins were reported to have superior surface characteristics (surface roughness [10,11], gloss [11]), flexural strength [12], and elastic modulus [13]), whereas 3D-printed denture base resins were reported to have better color stability [14] and fracture toughness [15] when compared to conventional denture base materials.

Denture wearers must clean their dentures to prevent the formation of biofilms on the denture surfaces. Brushing dentures using dentifrices can significantly affect the roughness and wear of both prosthetic and restorative materials [16]. As denture bases are in close proximity to oral tissues, it is vital that these materials have good surface properties. Dental prostheses with rough surfaces can affect the health of oral tissues because of the accumulation of microorganisms. As well as surface roughness, another major concern is the changes in color or staining of denture resins. Changes in the color of dental prostheses can make them appear damaged, which can lead to patient disappointment [17,18,19,20].

Color changes can occur because of impaired oral hygiene and can be further intensified by dietary and deleterious habits. Certain beverages such as coffee, tea, and wine as well as the use of tobacco can cause staining of acrylic resin [21]. Staining can be assessed either visually or by using instruments. Visual assessments are based on psychological and physiological perceptions, whereas the use of instruments can help to reduce the chances of errors or subjective assessments [22]. Spectrophotometers and colorimeters are frequently used to evaluate color changes in various dental materials [23].

Tobacco consumption is prevalent around the world and is consumed in various forms, including smoking tobacco (cigarettes, electronic cigarettes and heated tobacco products) and smokeless tobacco (khaini, mishri, toombak, tabbaq, betel quid, shammah, gutka, etc., which can be used as chewing tobacco, dry snuff and snus) [24,25,26]. Tobacco consumption is reported to be associated with the discoloration of teeth, as well as changes in the surface properties of dental materials [27,28,29,30]. Changes in color and surface roughness due to cigarette smoking are reported to be due to the deposition of various components such as tar, lead, carbon monoxide, nickel, etc., and the additional effect of the heat produced by the smoke [30,31,32]; whereas changes in color and surface roughness caused by smokeless tobacco can be due to the presence of various chemicals (formaldehyde, nicotine, polynuclear aromatic hydrocarbons, polonium 210, and N-nitrosamines), metals (cadmium, cyanide, arsenic, and benzene), and preservatives that affect the organic matrix of the dental materials [27,33]. Thus, the present study was conducted to evaluate the effects of two commercially available smokeless tobacco products on the color stability and surface roughness of denture base resins that were fabricated using three different techniques (CAD/CAM milling, 3D printing, and conventional heat polymerization). The tested null hypothesis was that there would be no significant changes in the color or surface roughness of the selected denture base materials when exposed to smokeless tobacco products.

## 2. Materials and Methods

The present in vitro comparative study was approved by the research board of the College of Dentistry, Jazan University (reference number: CODJU-2111I,09 November 2021). In the present study, the effects of two commercially available smokeless tobacco products on the color stability and surface roughness of denture base resins fabricated using three different techniques (CAD/CAM milling, 3D printing, and conventional heat polymerization) were evaluated and compared.

### 2.1. Materials

In this study, disk-shaped specimens were fabricated using three techniques and later immersed in either distilled water (control) or two different tobacco formulations (test groups) before being subjected to tests of the color stability and surface roughness. G*Power (version 3.1.9.7, 2020, Heinrich Heine University Düsseldorf, Düsseldorf, Germany) was used to estimate the sample sizes while assuming a nine-group comparison. The alpha was set at 0.05 with a power of 80%, and an effect size of 0.40 was used to calculate the sample size. A sample size of 126 (42 specimens in each group) was found to be sufficient.

### 2.2. Specimen Preparation

In total, 126 disk-shaped specimens (10 mm in diameter and 2 mm in thickness) were fabricated using three denture-fabrication techniques following the standard procedures for removable denture prosthesis (RDP) fabrication (*n* = 42 each): CAD/CAM subtraction (milling), CAD/CAM addition (3D printing), and conventional heat-polymerization compression molding (Table 1).

**Figure 1:**
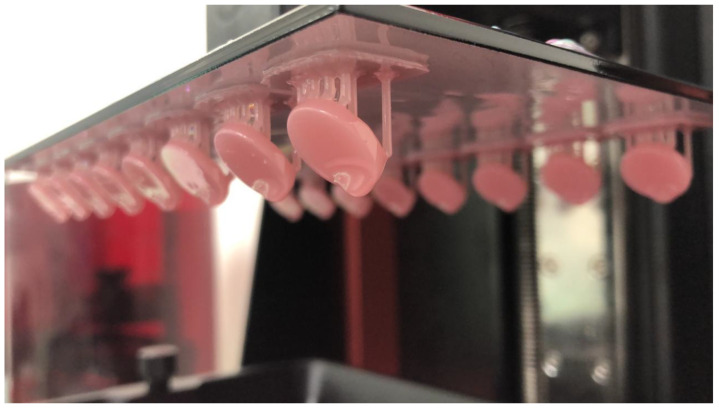
Specimens fabricated using 3D printing.

**Figure 2:**
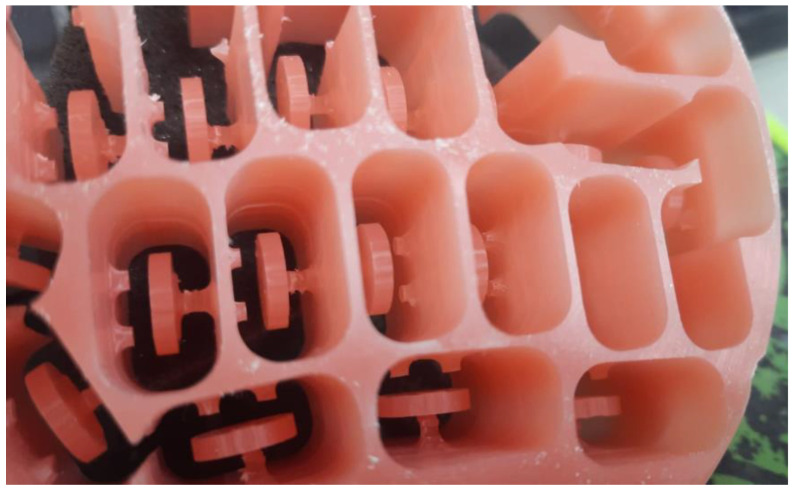
Specimens fabricated using CAD/CAM milling.

**Figure 3:**
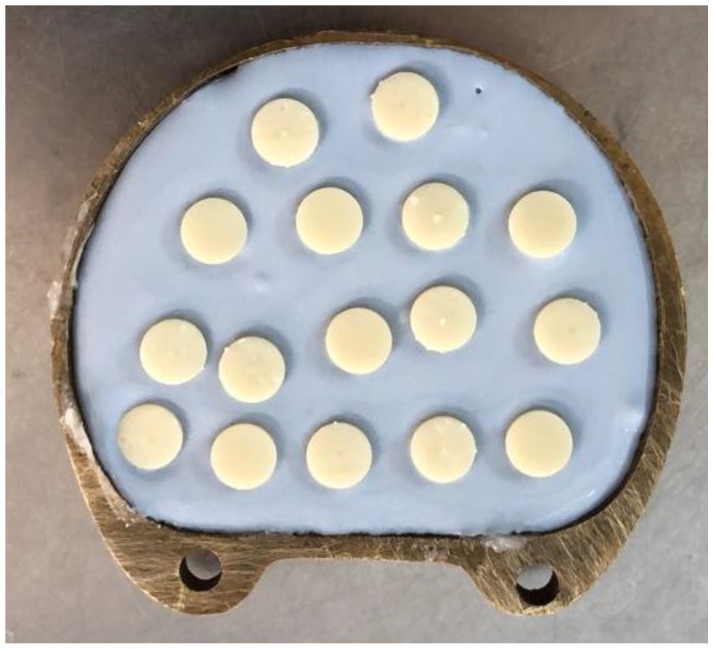
Wax patterns for fabricating specimens using the conventional compression-molding technique.

CAD software (Microsoft 3D builder, 16.0.2611.0, 2021, Microsoft Corporation, Redmond, WA, USA) was used for the virtual design of the disk-shaped specimens. The 3D-printing direction was set at 45 degrees, and a layer thickness of 50 μm was used to fabricate 42 specimens using a direct light processing (DLP) 3D printer (NextDent™ 5100, NextDent B.V., Soesterberg, The Netherlands) [10,14] (Figure 1). To fabricate the CAD/CAM-milled specimens, an Opera Pro-Expert 5 milling machine (Euromax Monaco, Monaco, Monaco) was used to dry mill a PMMA block (Figure 2). Additionally, the conventional compression-molding technique with a long curing cycle (74 °C for 8 h followed by 100 °C for 1 h) was used to fabricate another 42 specimens using a heat-polymerized acrylic resin (Figure 3) [10,14]. A MotoPol 8 grinder and polisher (Buehler GmbH, Dusseldorf, Germany) was used to finish one side of each specimen using wet abrasive carbide disks (Buehler Ltd., Lake Bluff, IL, USA) [10,14]. The specimens were then polished using polishing paste (Universal Polishing Paste, Ivoclar, Schaan, Liechtenstein). Dental loupes were used to check the quality of the final specimens, and poor-quality specimens were substituted with new samples. Only one surface of the specimens was polished to imitate a denture intaglio surface. The unpolished surfaces were coded using a low-speed handpiece, and the specimens were cleaned in an ultrasonic bath (Hygosonic, DURR Dental, Bietigheim-Bissingen, Germany) [10,14].

### 2.3. Initial Color and Surface-Roughness Testing

The samples were randomly divided into three subgroups (*n* = 14). They were dried using sterile tissue paper and their initial color (colorimeter, CS-10, Hangzhou Quality Lab Scientific Co. Ltd., Hangzhou, China) and surface roughness (3D optical non-contact surface profiler, UMT1, Campbell, CA, USA) were recorded. The color readings were recorded against a white background [34,35]. In total, three readings were recorded from the center of the specimens, and these values were then averaged. The CIELAB color system was used to describe the color coordinates.

### 2.4. Immersion Protocol

**Figure 4:**
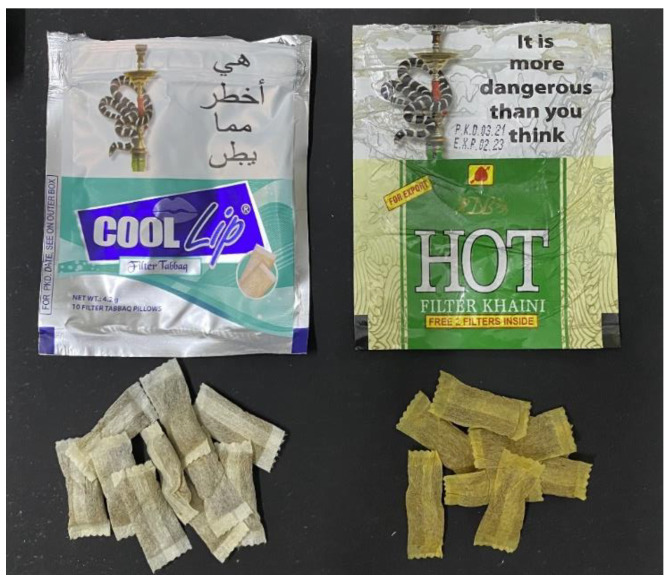
The smokeless tobacco (tabbaq and khaini) used in this study.

In this study, two common smokeless tobacco products were selected: khaini and tabbaq smokeless tobacco (Table 2 and Figure 4). Suspensions of these smokeless tobacco products were obtained with the help of the Substance Abuse and Toxicology Research Center, Jazan University. The solutions were prepared by mixing 20 pouches of smokeless tobacco in 50 mL of distilled water. In each group, three subgroups were created using 14 randomly selected specimens. The specimens from these subgroups were immersed in three different media (khaini smokeless tobacco, tabbaq smokeless tobacco, and artificial saliva) before being subjected to tests for color stability and surface roughness.

**Figure 5:**
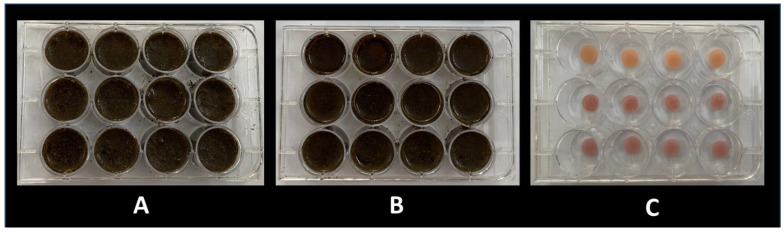
Specimens immersed in khaini smokeless tobacco (**A**), tabbaq smokeless tobacco (**B**), and artificial saliva (**C**).

**Figure 6:**
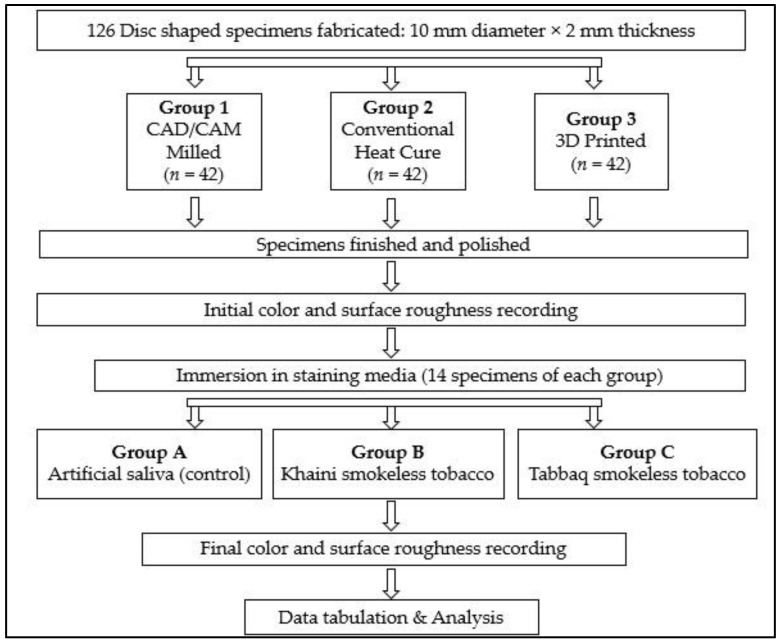
A flowchart depicting the study design.

The samples in the test groups were immersed in the smokeless tobacco solutions for 3 h at 37 °C (with the polished surface facing up). They were later washed in running water and immersed in an artificial salivary substitute solution at 37 °C for the rest of the day [36,37]. The control group specimens were immersed in artificial saliva for 24 h at 37 °C (Figure 5). This cycle was repeated for 90 days, following which the final color and surface-roughness measurements were recorded (Figure 6).

### 2.5. Final Color and Surface-Roughness Testing

For the final color and surface-roughness readings, measurements were taken using the same protocol as that for the initial color and surface-roughness readings. The CIELAB color-difference formula was used to calculate the changes in color as shown in Equation (1) [38,39]:

ΔE*ab = [(ΔL*)^2^ + (Δa*)^2^ + (Δb*)^2^ ]^1/2^ (1)

where ΔL*, Δa*, and Δb* represent the differences in the L* (lightness/darkness value), a* (red/green axis), and b* (yellow/blue axis) values, respectively, before and after immersion in the selected media.

The changes in surface roughness (µm) were calculated using Equation (2). All measurements were recorded by a single trained operator, and blinding was adopted to avoid bias.

ΔRa = fRa − iRa(2)

where iRa is the initial surface roughness measurement and fRa is the final surface roughness measurement after immersion in the tested solutions.

### 2.6. Data Analysis

The data were collected and tabulated using a Microsoft Excel spreadsheet (version 1910, 2019, Microsoft Inc., Redmond, WA, USA), and a statistical analysis was performed using SPSS version 24.0 (IBM SPSS Statistics for Windows, Version 24.0, 2016, IBM Corp., Armonk, NY, USA). The mean color and surface roughness changes were compared between all groups and between the subgroups of each group using Two-way ANOVA test.

## 3. Results

**Figure 7:**
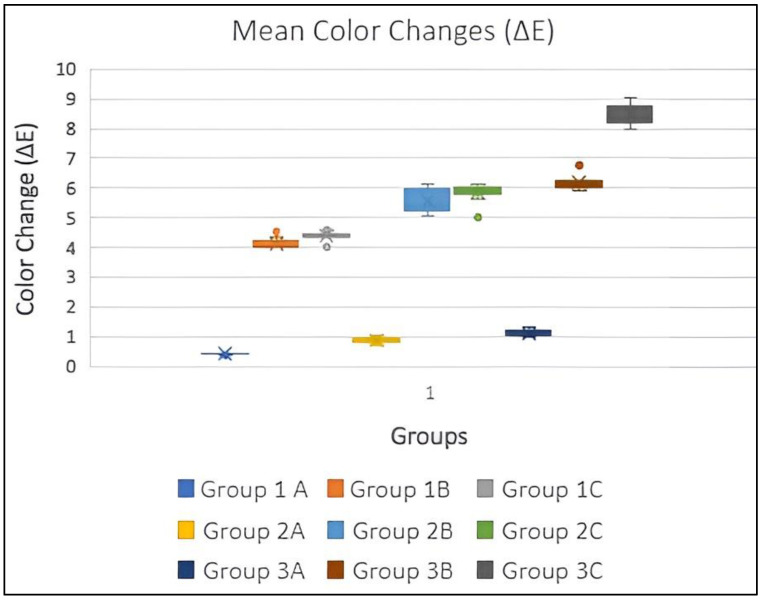
Mean color changes (ΔE) in all groups.

**Figure 8:**
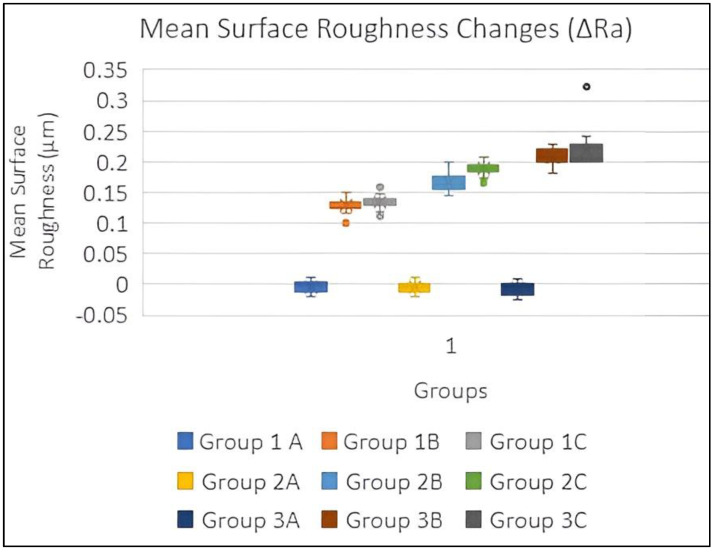
Mean surface roughness changes (ΔRa) in all groups.

The mean color changes (ΔE) were recorded for all the study groups. We observed that the color changes were greatest after tabbaq usage in all study groups, followed by khaini usage and artificial saliva. The greatest color changes were observed in 3D-printed specimens, followed by heat-polymerized and milled specimens. This meant that the greatest color changes were observed in the case of dentures that were prepared using 3D printing and were then exposed to tabbaq (Table 3 and Figure 7).The mean changes in surface roughness (ΔRa) were recorded in all study groups. We observed that the surface roughness readings were highest after tabbaq usage in all study groups, followed by khaini usage and artificial saliva. The surface roughness readings were highest among samples prepared using 3D printing, followed by heat polymerization and milling (Table 3 and Figure 8). A two-way ANOVA was used to check the difference between groups, subgroups, and interactions between groups and subgroups. A statistically significant difference was found between groups and also between subgroups for color change and surface roughness change. There was also a significant interaction between groups and subgroups.

The Bonferroni post hoc test was used to check differences between color change (ΔE) and surface roughness change (ΔRa) between groups and subgroups, as shown in Table 4 and Table 5. There was a significant difference between all three groups and all the three subgroups for both the parameters of color change (ΔE) and surface roughness change (ΔRa). Statistically significant (*p*-value < 0.05) correlations were observed between all study groups after immersion in the three different suspensions. This meant that tabbaq caused the greatest changes in color and surface roughness and that the effect was highest in 3D-printed samples, followed by heat-polymerized and milled specimens.

## 4. Discussion

The standard that is used to measure the clinical success of any aesthetic material for prostheses is color stability. Thus, the evaluation of changes in color using various color measuring devices such as spectrophotometers is quite frequent nowadays. Various studies [29,31,32,40,41] have assessed changes in denture parameters such as color and surface texture due to the effects of certain beverages and smoking, but studies on the effects of different tobacco products on recently introduced denture bases are lacking. Thus, we conducted this study to assess the effects of two different smokeless tobacco products on denture bases that were prepared using three different techniques. This study not only evaluated the effects of tobacco but also assessed the best fabrication technique in terms of the color stability and surface quality. In our study, we observed that the mean color and surface-roughness changes were significantly higher after tabbaq usage in all study groups followed by khaini usage and artificial saliva; the greatest changes were observed in the 3D-printed specimens followed by the heat-polymerized and milled specimens.

Changes in the surface roughness of dental materials can alter their translucency and opacity, thus affecting their color and altering their appearance [42]. Al Moaleem et al. [29] observed that smokeless tobacco had the capability to change the color of dental ceramics, but this change was statistically insignificant. The presence of a highly polished glaze layer on dental ceramics increased their color stability. In the current study, significant changes in color in the tested polymeric resins could have been due to their porous structure and less polished outer surface when compared to dental ceramics. Other studies have reported that certain habits such as tobacco chewing or cigarette smoking can lead to the significant discoloration of teeth, composites, and denture reline materials [27,30,43,44]. The extent of color change can be managed by minimizing the deposits from tobacco and cigarette smoking. Similarly, Willeres et al. [45] also observed that tobacco use could cause high mean color changes. In contrast, no changes in color were reported by Prajapati et al. [46] when ceramic restorative materials were exposed to tobacco extracts and cigarette smoking. Various uninterrupted deleterious habits such as consuming different forms of tobacco can lead to an increase in the retention of color over the denture base material.

The results of our study were in agreement with the results of the studies by Yao et al. [47] and Tas¸ın et al. [48], who found that CAD/CAM-milled resin-based interim fixed partial denture specimens had a better color stability than 3D-printed resins when immersed in different staining solutions. Shin et al. [49] evaluated the changes in color of 3D-printed and CAD/CAM-milled interim fixed partial denture resins after immersing them in colorant for 7 days or more and reported that the color change for the 3D-printed resins was above the acceptable threshold (∆E > 2.25).

Dayan et al. [4] evaluated the color stability of denture bases fabricated using different techniques (heat polymerization, auto-polymerization, light activation, and CAD/CAM milling) after immersing the bases in different storage media and reported that the CAD/CAM-milled denture base resins displayed the highest color stability compared to the other tested denture base resins. Al-Qarni et al. [50] found that milled acrylic resins had a poor stainability compared to conventional resin materials. CAD/CAM-milled denture bases with teeth and acrylic resin denture bases have shown higher stain resistance at the tooth–denture base interface compared to denture bases fabricated using the conventional methods of processing [4]. Abdulallah et al. [51] found that significant improvements were observed in CAD/CAM acrylic resins in terms of the adaptation, surface hardness, and roughness of denture bases compared to conventional and 3D-printed acrylic resins.

Studies have reported that ΔE values above 3.3 are considered clinically perceptible: ΔE values between 3.3–5.0 are considered clinically acceptable, whereas values above 5 are considered clinically unacceptable [35,52,53]. In the present study, the ΔE values obtained for all three tested denture base resins after immersion in both smokeless tobacco solutions were above the clinically perceptible limits For the CAD/CAM-milled denture base resins, the ΔE values were within the clinically acceptable range (4.13 and 4.38), while those for conventional heat-polymerized resins (5.54 and 5.85) and 3D-printed resins (6.2 and 8.46) were above the clinically acceptable range.

Mahross et al. [28], Sayed et al. [30], and Alandia-Roman et al. [54] reported changes in the surface roughness of tested materials (denture relining materials, denture base materials, and dental composites, respectively) after they were exposed to cigarette smoke. The acceptable range of surface roughness was 0.2 µm as documented in the literature [55]. An increase in surface roughness will cause more plaque accumulation and may lead to mucositis and irritation of the soft tissues. In the current study, only the 3D-printed resins displayed changes in surface roughness that were higher than the acceptable threshold (0.2081 and 0.2191 µm) when immersed in the smokeless tobacco solutions. The values for the conventional polymerized (0.1655 and 0.1884) and CAD/CAM-milled resins (0.1289 and 0.1349 µm) were below the acceptable range.

These findings could be due to the fact that CAD/CAM milled resins are manufactured industrially and have high polymerization and cross-linking compared to conventional heat-polymerized and 3D-printed resins; thus, they have been reported to have a better color stability and cause smaller changes in the surface roughness [14,56,57,58,59,60,61,62,63]. The high polarity of 3D-printed resins due to the hydrophilic monomer, the lack of filler particles, the presence of uncured layers, the quantity of the residual monomer, and the high water sorption could be additional causes of their high stainability and increased surface roughness [48,64,65,66,67,68,69]. In the case of conventional acrylic resins, they have been found to be susceptible to staining due to their inherent water sorption property, which causes water and color absorption. These absorbed molecules of water further cause a softening of resin components by acting as plasticizers, thereby leading to the expansion of the polymer matrix via the separation of the polymer chains, which in turn leads to the penetration of the staining solutions and the discoloration of the resin [70,71].

Higher degrees of cross-linking among different resin bases lead to increased stability against color and surface roughness changes. The choice of an appropriate denture base material is critical for patients who consume tobacco in any form. Patient education and awareness are important to achieve successful treatment outcomes.

### Limitations of the Study

1. The dental materials in the oral cavity are exposed to various staining food components, temperature changes, pH changes, and occlusal loading. The effect of these additional factors could not be replicated in the current study. Future studies should be planned to evaluate the effect of smokeless tobacco on denture bases worn by patients.
2. The effect of smokeless tobacco on denture base materials was evaluated only for 90 days. More studies with a longer duration should be conducted.
3. Only one surface of the specimens was polished to imitate to denture intaglio surface, but an unpolished surface could influence the color measurements.
4. Future research should evaluate the combined effects of denture cleansers, mechanical denture cleansing techniques and the different types of smokeless tobacco on denture base materials.
5. The current study evaluated only two forms of smokeless tobacco. The effect of other forms of smokeless tobacco on different physical and mechanical properties of denture base materials should be evaluated in future studies.

## 5. Conclusions

Within the limitations of the present in vitro study, we concluded that the mean changes in the color and surface roughness were significantly higher in dentures that were prepared using 3D printing followed by those that were prepared using heat polymerization and CAD/CAM milling. In all study groups, the greatest changes were found when the denture base resins were immersed in the tabbaq smokeless tobacco followed by the khaini smokeless tobacco and the artificial saliva.

## Figures and Tables

**Table 1:** Details of the denture base resins used in this study.

Group	Manufacturing Technique	Commercial Product and Manufacturer	Main Ingredient
1	CAD/CAM milling	Wieland; Wieland Dental +, Technik GmbH and Co. KG, Pforzheim, Germany	PMMA resin
2	Heat polymerization using the conventional compression-molding technique	Meliodent; Kulzer-GmbH, Hanau, Germany	PMMA resin
3	3D printing	NextDent; Denture 3D+, Vertex-Dental, Soesterberg, the Netherlands	Dimethacrylate-based resins

**Table 2:** Details of the immersion solutions utilized in this study.

Sub Group	Immersion Solution	Trade Name	Composition	pH
A	Artificial salivary substitute (control)	-	Distilled water, NaCl, KCl, and CaCI_2_	7.4
B	Khaini smokeless tobacco	HOT Filter Khaini (SUN Products, Gujarat, India)	Tobacco, lime water, menthol, and spices	9.3
C	Tabbaq smokeless tobacco	Cool Lips Filter Tabbaq (Tej Ram Dharam Paul, Gujarat, India.)	Tobacco, mint, and spices (such as cinnamon, cardamom, and cloves)	9.4

**Table 3:** Mean color changes (ΔE) and surface roughness changes (ΔRa) in all groups.

Groups	Subgroups	*n*	Color Change (ΔE)		Surface Roughness Change (ΔRa)	
			Mean	SD	Mean	SD
Group 1 (CAD/CAM Milled)	GROUP 1A (Artificial Saliva)	14	0.418	0.022	−0.003	0.011
	GROUP 1B (Khaini)	14	4.129	0.165	0.129	0.013
	GROUP 1C (Tabbaq)	14	4.383	0.144	0.135	0.012
	Total	42	2.977	1.838	0.087	0.066
Group 2 (Heat-Polymerized)	GROUP 2A (Artificial Saliva)	14	0.888	0.087	−0.005	0.010
	GROUP 2B (Khaini)	14	5.544	0.412	0.166	0.015
	GROUP 2C (Tabbaq)	14	5.851	0.274	0.188	0.011
	Total	42	4.094	2.315	0.116	0.088
Group 3 (3D-Printed)	GROUP 3A (Artificial Saliva)	14	1.118	0.122	−0.007	0.011
	GROUP 3B (Khaini)	14	6.204	0.274	0.208	0.015
	GROUP 3C (Tabbaq)	14	8.457	0.375	0.219	0.033
	Total	42	5.260	3.118	0.140	0.108
Total	Artificial Saliva	42	0.808	0.307	−0.005	0.011
	Khaini	42	5.292	0.924	0.168	0.036
	Tabbaq	42	6.230	1.727	0.181	0.041
	Total	126	4.110	2.633	0.114	0.091
2-Way ANOVA: Group Differences			*p* < 0.001		*p* < 0.001	
2-Way ANOVA: Subgroup Differences			*p* < 0.001		*p* < 0.001	
2-Way ANOVA: Group–Subgroup Interaction			*p* < 0.001		*p* < 0.001	

**Table 4:** Comparison between groups and subgroups to check differences in color change (ΔE) using post hoc Bonferroni test.

Multiple Comparisons (Group)							
(I) Group		Mean Difference (I-J)	Std. Error	Sig.	95% Confidence Interval		
					Lower Bound	Upper Bound	
CAD/CAM Milled	Heat-Polymerized	−1.1177 *	0.05304	0.000	−1.2465	−0.9889	*p* < 0.001
	3D-Printed	−2.2830 *	0.05304	0.000	−2.4118	−2.1541	*p* < 0.001
Heat-Polymerized	CAD/CAM Milled	1.1177 *	0.05304	0.000	0.9889	1.2465	*p* < 0.001
	3D-Printed	−1.1653 *	0.05304	0.000	−1.2941	−1.0364	*p* < 0.001
3D-Printed	CAD/CAM Milled	2.2830 *	0.05304	0.000	2.1541	2.4118	*p* < 0.001
	Heat-Polymerized	1.1653 *	0.05304	0.000	1.0364	1.2941	*p* < 0.001
Multiple Comparisons (Subgroup)							
Artificial Saliva	Khaini	−4.4840 *	0.05304	0.000	-4.6129	−4.3552	*p* < 0.001
	Tabbaq	−5.4219 *	0.05304	0.000	-5.5508	−5.2931	*p* < 0.001
Khaini	Artificial Saliva	4.4840 *	0.05304	0.000	4.3552	4.6129	*p* < 0.001
	Tabbaq	−0.9379 *	0.05304	0.000	-1.0667	−0.8091	*p* < 0.001
Tabbaq	Artificial Saliva	5.4219 *	0.05304	0.000	5.2931	5.5508	*p* < 0.001
	Khaini	−0.9379 *	0.05304	0.000	0.8091	1.0667	*p* < 0.001

Based on observed means. The error term is mean square (error) = 0.059. * The mean difference is significant at the 0.05 level.

**Table 5:** Comparison between groups and subgroups to check differences in surface roughness change (ΔRa) using post hoc Bonferroni test.

Multiple Comparisons (Group)							
(I) Group		Mean Difference (I-J)	Std. Error	Sig.	95% Confidence Interval		
					Lower Bound	Upper Bound	
CAD/CAM Milled	Heat-Polymerized	−0.0295 *	0.00353	0.000	−0.0381	−0.0209	*p* < 0.001
	3D-Printed	−0.0532 *	0.00353	0.000	−0.0618	−0.0447	*p* < 0.001
Heat-Polymerized	CAD/CAM Milled	0.0295 *	0.00353	0.000	0.0209	0.0381	*p* < 0.001
	3D-Printed	−0.0237 *	0.00353	0.000	−0.0323	−0.0152	*p* < 0.001
3D-Printed	CAD/CAM Milled	0.0532 *	0.00353	0.000	0.0447	0.0618	*p* < 0.001
	Heat-Polymerized	0.0237 *	0.00353	0.000	0.0152	0.0323	*p* < 0.001
Multiple Comparisons (Subgroup)							
Artificial Saliva	Khaini	−0.1725 *	0.00353	0.000	−0.1810	−0.1639	*p* < 0.001
	Tabbaq	−0.1858 *	0.00353	0.000	−0.1943	−0.1772	*p* < 0.001
Khaini	Artificial Saliva	0.1725 *	0.00353	0.000	0.1639	0.1810	*p* < 0.001
	Tabbaq	−0.0133 *	0.00353	0.001	−0.0218	−0.0047	*p* = 0.001
Tabbaq	Artificial Saliva	0.1858 *	0.00353	0.000	0.1772	0.1943	*p* < 0.001
	Khaini	0.0133 *	0.00353	0.001	0.0047	0.0218	*p* = 0.001

Based on observed means. The error term is mean square (error) = 0.000. * The mean difference is significant at the 0.05 level.

## Data Availability

The data that supported the findings of this study are available from the corresponding author upon reasonable request.
